# Recruitment pattern of the diaphragm and extradiaphragmatic inspiratory muscles in response to different levels of pressure support

**DOI:** 10.1186/s13613-020-00684-6

**Published:** 2020-05-29

**Authors:** L. H. Roesthuis, J. G. van der Hoeven, H. W. H. van Hees, W.-J. M. Schellekens, J. Doorduin, L. M. A. Heunks

**Affiliations:** 1 Department of Intensive Care Medicine, Radboud University Medical Center, Nijmegen, The Netherlands; 2 Department of Pulmonary Diseases, Radboud University Medical Center, Nijmegen, The Netherlands; 3 Department of Anesthesiology, UMC Utrecht, Utrecht, The Netherlands; 4 Donders Institute for Brain, Cognition and Behaviour, Department of Neurology, Radboud University Medical Center, Nijmegen, The Netherlands; 5 Department of Intensive Care Medicine, Amsterdam UMC, Location VUmc, Postbox 7057, 1007 MB Amsterdam, The Netherlands

**Keywords:** Ventilated critically ill patients, Respiratory drive, Electrical activity of the diaphragm, Extradiaphragmatic inspiratory muscle activity, Surface electromyography

## Abstract

**Background:**

Inappropriate ventilator assist plays an important role in the development of diaphragm dysfunction. Ventilator under-assist may lead to muscle injury, while over-assist may result in muscle atrophy. This provides a good rationale to monitor respiratory drive in ventilated patients. Respiratory drive can be monitored by a nasogastric catheter, either with esophageal balloon to determine muscular pressure (gold standard) or with electrodes to measure electrical activity of the diaphragm. A disadvantage is that both techniques are invasive. Therefore, it is interesting to investigate the role of surrogate markers for respiratory dive, such as extradiaphragmatic inspiratory muscle activity. The aim of the current study was to investigate the effect of different inspiratory support levels on the recruitment pattern of extradiaphragmatic inspiratory muscles with respect to the diaphragm and to evaluate agreement between activity of extradiaphragmatic inspiratory muscles and the diaphragm.

**Methods:**

Activity from the alae nasi, genioglossus, scalene, sternocleidomastoid and parasternal intercostals was recorded using surface electrodes. Electrical activity of the diaphragm was measured using a multi-electrode nasogastric catheter. Pressure support (PS) levels were reduced from 15 to 3 cmH_2_O every 5 min with steps of 3 cmH_2_O. The magnitude and timing of respiratory muscle activity were assessed.

**Results:**

We included 17 ventilated patients. Diaphragm and extradiaphragmatic inspiratory muscle activity increased in response to lower PS levels (36 ± 6% increase for the diaphragm, 30 ± 6% parasternal intercostals, 41 ± 6% scalene, 40 ± 8% sternocleidomastoid, 43 ± 6% alae nasi and 30 ± 6% genioglossus). Changes in diaphragm activity correlated best with changes in alae nasi activity (*r*^2^ = 0.49; *P* < 0.001), while there was no correlation between diaphragm and sternocleidomastoid activity. The agreement between diaphragm and extradiaphragmatic inspiratory muscle activity was low due to a high individual variability. Onset of alae nasi activity preceded the onset of all other muscles.

**Conclusions:**

Extradiaphragmatic inspiratory muscle activity increases in response to lower inspiratory support levels. However, there is a poor correlation and agreement with the change in diaphragm activity, limiting the use of surface electromyography (EMG) recordings of extradiaphragmatic inspiratory muscles as a surrogate for electrical activity of the diaphragm.

## Background

Diaphragm dysfunction frequently develops in critically ill patients [1–3]. Among other factors, inappropriate ventilator assist plays a prominent role in the pathogenesis. Ventilator under-assist may lead to muscle injury, while over-assist may result in muscle atrophy [4–9]. This provides a good rationale to monitor respiratory effort in ventilated Intensive Care Unit (ICU) patients [10–13]. Calculation of muscular pressure (Pmus) based on changes in esophageal pressure and chest wall elastic recoil pressure is considered the gold standard to monitor respiratory effort [14, 15]. Electrical activity of the diaphragm (EAdi), acquired with a nasogastric catheter with multiple electrodes, has been used to quantify respiratory effort as well [10–12]. Although electromyography (EMG) does not directly reflect effort, a linear correlation between Pmus and EAdi has been reported [16]. However, both techniques are invasive and the multi-electrode esophageal catheter is only available with one specific ventilator (Servo-i/u). However, EAdi can also be obtained using surface electrodes [17, 18], although with specific challenges, especially in obese patients, or after abdominal surgery. Therefore, it is of interest to investigate surrogate markers for respiratory drive, such as activity of extradiaphragmatic inspiratory muscles [19].

The extradiaphragmatic inspiratory muscles support the diaphragm to maintain adequate ventilation, but each muscle has specific other tasks. For instance, the alae nasi and genioglossus maintain upper airway patency [20–22], the parasternal intercostals stabilize the chest wall and facilitate rotation of the trunk [23, 24], while the scalene and sternocleidomastoid are involved in rotation of the head and flexion of the neck [25]. In previous studies, surface EMG has been used in ventilated ICU patients to evaluate activity of the extradiaphragmatic inspiratory muscles, including alae nasi, parasternal intercostals [26], scalene [26, 27], sternocleidomastoid and genioglossus [26–28]. Overall, these papers concluded that monitoring of respiratory drive in ventilated ICU patients by surface EMG is feasible and useful. However, the effect of different levels of ventilator support on the relation between diaphragmatic and extradiaphragmatic inspiratory muscle activity was not studied in detail, as well as a comparison on individual patient level between activity of the diaphragm and extradiaphragmatic inspiratory muscles.

Therefore, the aim of the current study is to investigate in invasively ventilated ICU patients the recruitment pattern of extradiaphragmatic inspiratory muscles with respect to the diaphragm in response to different inspiratory support levels and to evaluate agreement between activity of the extradiaphragmatic inspiratory muscles and the diaphragm.

## Methods

### Study population

This study was conducted in a mixed ICU of the Radboud University Medical Center. Adult patients mechanically ventilated for at least 3 days, with a NAVA catheter (Maquet Critical Care, Sölna, Sweden) in situ and inspiratory support ≤ 10 cmH_2_O, positive end-expiratory pressure (PEEP) level ≤ 10 cmH_2_O and FiO_2_ < 0.60, were recruited. Exclusion criteria were hemodynamic instability [i.e. systolic blood pressure < 100 mmHg; heart rate < 50 or > 120 beats/min and use of high-dose vasopressors (i.e. norepinephrine > 0.2 µg/kg/min)]. Based on previous physiological studies from our group [29–32], a convenience sample of 17 patients was considered appropriate. The institutional review board waived informed consent as risks associated with this study were negligible. Patients or patient surrogate decision-makers were informed about the study purpose and design.

### Study protocol

This was a prospective clinical study. All patients were ventilated with a Servo-i ventilator (Maquet Critical Care, Sölna, Sweden). Activity from the alae nasi, genioglossus, scalene, sternocleidomastoid and parasternal intercostals was continuously measured as described below. Pressure support (PS) level was reduced every 5 min with steps of 3 cmH_2_O, from 15 cmH_2_O to 3 cmH_2_O. The duration of 5 min for each study step was considered appropriate as it has been shown previously that respiratory drive adapts within 5 min of altered loading [30, 33, 34]. PEEP, inspiratory rise time, cycle-off criteria and trigger sensitivity were maintained as dictated by clinical protocol.

### Data acquisition

Flow was acquired by placing a single use flow sensor (Hamilton Medical AG, Bonaduz, Switzerland) between the endotracheal tube and Y-piece of the ventilator circuit, connected to a pressure transducer (range ± 50 kPa, Freescale Semiconductor, Tempe, AZ, USA). The pressure transducer was connected to the auxiliary channel (1.4 μV/bit, amplification factor: 1) of the Porti 24 data acquisition system (22 bits, TMSi; The Netherlands).

EAdi was obtained using a multi-electrode nasogastric catheter. Correct positioning of the catheter was obtained using standard software supplied with the ventilator by the manufacturer. Electrical activity from the extradiaphragmatic muscles was recorded using wet gel silver–silverchloride surface electrodes, 30 × 22 mm in diameter (Ambu^®^ Blue sensor N, Ballerup, Denmark). Recording locations were cleaned to improve signal to noise ratio. For the alae nasi, electrodes were placed on each side of the nose. For the genioglossus electrodes were placed just below the chin and above the hyoid bone. Electrode placement of the sternocleidomastoid and scalene was guided by ultrasonography and electrodes were placed in the lower thirds of the muscles [35]. For the parasternal intercostals, the active electrode was placed in the second intercostal space 3 cm lateral from the sternum and the reference on an adjacent rib. A ground electrode was placed on the patient’s wrist. Visual feedback for all (EMG) signals was available.

EAdi was acquired with a Porti 16 data acquisition system (22 bits, TMSi; The Netherlands) with unipolar electrophysiological channels (71.5 nV/bit, amplification factor: 20). EMG from extradiaphragmatic muscles was acquired with a Porti 24 data acquisition system (22 bits, TMSi; The Netherlands) with bipolar electrophysiological channels (71.5 nV/bit, amplification factor: 20). Flow and EMG signals were digitized with a sample frequency of 2048 Hz and stored synchronously on a hard disk using Portilab (TMSi; The Netherlands). Offline analysis was performed with Matlab (R2014b, The Mathworks, Natick, Massachusetts, USA).

### Offline signal processing

EAdi was processed as described previously [30]. Surface EMG signals were band-pass filtered using a 25–500 Hz second-order Butterworth filter. If present, ECG artifacts were removed from the EMG using a wavelet-based adaptive filter [36]. From each recording (i.e. from every muscle and PS level) at least 10 breaths free of artifacts were selected at the end of each period. For each period the root mean square of the EMG was determined with a time averaging period of 2 ms. Further smoothing was obtained by applying a moving average filter with a window size of 200 ms to obtain a mean EMG envelope. An example of processing of the EMG signal is given in Additional file 1: Figure S1. Inspiratory efforts were detected from the flow signal and segmented in epochs time-locked to the inspiratory efforts. These epochs started 1 s before the inspiratory effort and terminated 1.5 s after the onset of the inspiratory effort [37].

### Parameter calculation

Respiratory rate, tidal volume, minute ventilation and inspiratory time were calculated from the flow signal. A threshold was determined visually from the mean EMG envelops to detect onset, peak and end of muscle activity (Additional file 1: Figure S1). The maximal amplitude of muscle activity during inspiration was defined as EMG_peak_. In addition, area under the curve of muscle EMG activity was calculated from onset of muscle activity till muscle activity was reduced to 70% of EMG_peak_, multiplied with respiratory rate (EMG_AUC/min_). Both parameters were normalized to muscle activity at pressure support level 3 cmH_2_O (% PS3). Timing of onset, peak and end of muscle activity were calculated relatively to the onset time of EAdi (Additional file 1: Figure S1).

### Statistical analysis

To compare the magnitude and timing of respiratory muscle activity between PS levels, one-way analysis of variance for repeated measures was performed (Friedman test). Post-hoc analysis was performed with Dunn’s Multiple Comparison test, to correct for multiple comparisons. Repeated observation analysis was performed to investigate whether changes in diaphragm activity would also result in the same changes in extradiaphragmatic inspiratory muscle activity [38]. EAdi and surface EMG for each of the extradiaphragmatic inspiratory muscles were compared using Bland–Altman analysis. For all tests, a two-tailed *P* < 0.05 was considered significant. Data are presented as mean ± standard error of the mean (SEM) for parametric data or median (interquartile range) for non-parametric data. Statistical analyses were performed with Prism 5 (Graphpad software, San Diego, CA, USA).

## Results

Seventeen patients with a NAVA catheter for clinical reasons were consecutively enrolled. Mean body mass index was 25.9 ± 1.7 kg/m^2^. Other patient characteristics are shown in Table 1. No adverse events were reported during the study.

**Table 1:** Patient characteristics

Subject	Age (years)	Gender	Reason of admission	Use of sedation	Baseline ventilator settings		Days of MV	RASS	pH	PaCO_2_ (mmHg)
					PS level (cmH_2_O)	PEEP level (cmH_2_O)				
1	65	M	Postoperative after AAA	Haloperidol, sufentanil, midazolam	10	10	8	− 1	7.41	37.5
2	64	F	COPD Gold I	Haloperidol	10	8	27	0	7.43	51.0
3	70	M	Bilateral pneumonia	Haloperidol	10	6	22	− 2	7.42	58.5
4	68	M	Hypoxemia, suspected aeCOPD	Haloperidol	10	5	28	0	7.49	40.5
5	67	M	CABG	Haloperidol, dexmedetomidine, sufentanil	10	10	21	0	7.50	41.3
6	71	M	COPD Gold III	Oxazepam	10	5	45	0	7.45	64.5
7	69	M	CABG, PJP	–	8	6	11	− 1	7.42	58.5
8	68	M	Pneumonia, COPD Gold III	Haloperidol, sufentanil	10	5	3	− 3	7.48	32.3
9	65	M	CABG	Haloperidol, sufentanil	10	6	19	0	7.52	36.0
10	46	M	Bentall procedure	Midazolam, sufentanil	10	8	3	− 4	7.36	38.3
11	62	M	Septic profile, ARDS	Haloperidol, oxazepam, sufentanil	10	6	15	NA	7.44	35.3
12	59	F	COPD Gold II	Remifentanil	10	5	7	NA	7.43	42.8
13	65	F	aeCOPD Gold III	Midazolam, sufentanil	10	5	8	− 2	7.48	51.0
14	75	F	Hypercapnic coma, weaning problems	–	0	10	20	0	7.42	50.3
15	52	M	Mediastinitis, COPD Gold II	Haloperidol	0	8	35	− 2	NA	NA
16	69	M	aeCOPD Gold III	Haloperidol, midazolam, sufentanil	10	5	3	− 2	7.45	42.0
17	47	F	Weaning problems	–	8	5	20	0	7.41	67.5

*MV* mechanical ventilation, *RASS* Richmond Agitation-Sedation Scale, *PS* pressure support, *PEEP* positive end-expiratory pressure, *M* male, *F* female, *AAA* abdominal aortic aneurysm, *COPD* chronic obstructive pulmonary disease, *CABG* coronary artery bypass graft, *PJP* pneumocystic jirovecii pneumonia, *ARDS* acute respiratory distress syndrome, *NA* not available

### Response of the extradiaphragmatic inspiratory muscles to different levels of inspiratory support

**Fig. 1:**
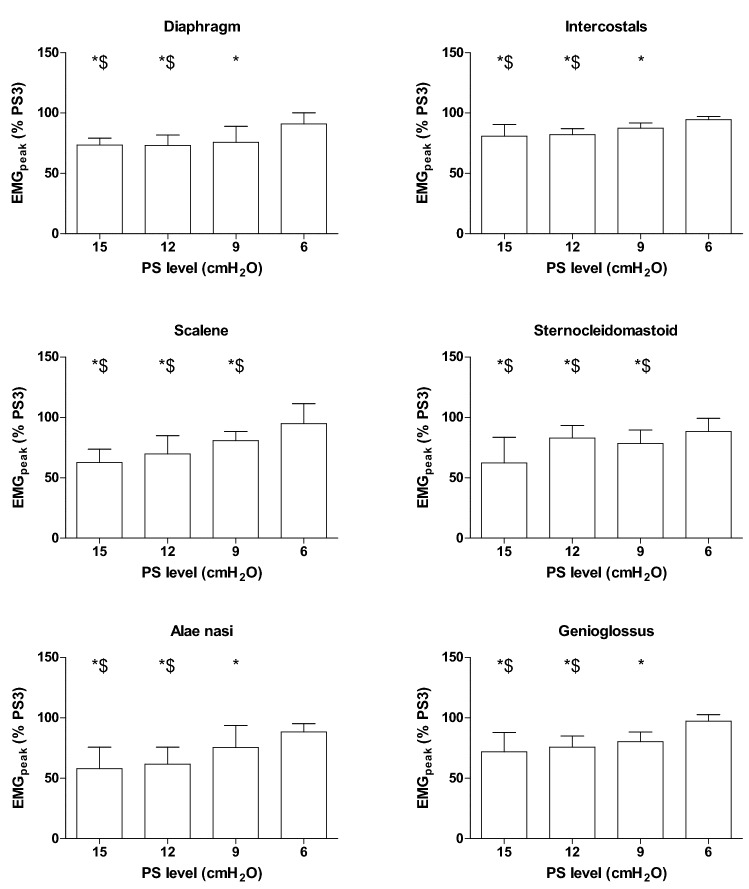
Extradiaphragmatic inspiratory muscle activity increases when lower support levels are applied (*P* < 0.05). The peak electromyography (EMG_peak_) values are normalized to activity at 3 cmH_2_O pressure support (PS). Values are represented as median (interquartile range). The number of subjects in which EMG parameters could be determined differed per muscle and PS level. Globally, for the alae nasi *N* = 14, genioglossus *N* = 10, scalene *N* = 16, sternocleidomastoid and diaphragm *N* = 17, and parasternal intercostals *N* = 15. *Significant difference with PS 3, ^$^significant difference with PS 6 (*P* < 0.05)

Reducing inspiratory PS level increased respiratory frequency, and decreased tidal volume and minute ventilation (Table 2). PS level did not affect inspiratory time (Table 2). As expected, EAdi_peak_ increased while lowering PS level (Fig. 1). In general, the extradiaphragmatic inspiratory muscles EMG_peak_ followed a similar pattern (Fig. 1), although the responses varied strongly among muscles and patients (Additional file 1: Figure S2). EMG_AUC/min_ provided similar results (Additional file 1: Figure S3). In several patients, extradiaphragmatic inspiratory muscle activity could not be detected: alae nasi (*N* = 3), genioglossus (*N* = 7), scalene (*N* = 1) and parasternal intercostal muscles (*N* = 2).

**Table 2:** Ventilatory parameters at different support levels

	PS 15	PS 12	PS 9	PS 6	PS 3
Tidal volume/kg (mL/kg)	7.2 ± 0.6	6.6 ± 0.6	6.2 ± 0.5*	6.1 ± 0.5*	5.8 ± 0.4*^$^
Respiratory rate (breaths/min)	24 ± 2	25 ± 2	27 ± 2	27 ± 2*	27 ± 2*
Minute ventilation (L)	10.9 ± 0.8	10.6 ± 0.8	10.5 ± 0.9	10.6 ± 1.0	10.3 ± 0.9*
Inspiratory time (s)	0.86 ± 0.07	0.83 ± 0.06	0.83 ± 0.07	0.86 ± 0.07	0.84 ± 0.06

Values are represented as mean ± SEM

*PS* pressure support level

*Significant difference with PS 15, ^$^significant difference with PS 12 (P < 0.05)

### Relationship between EAdi and extradiaphragmatic inspiratory EMG

**Fig. 2:**
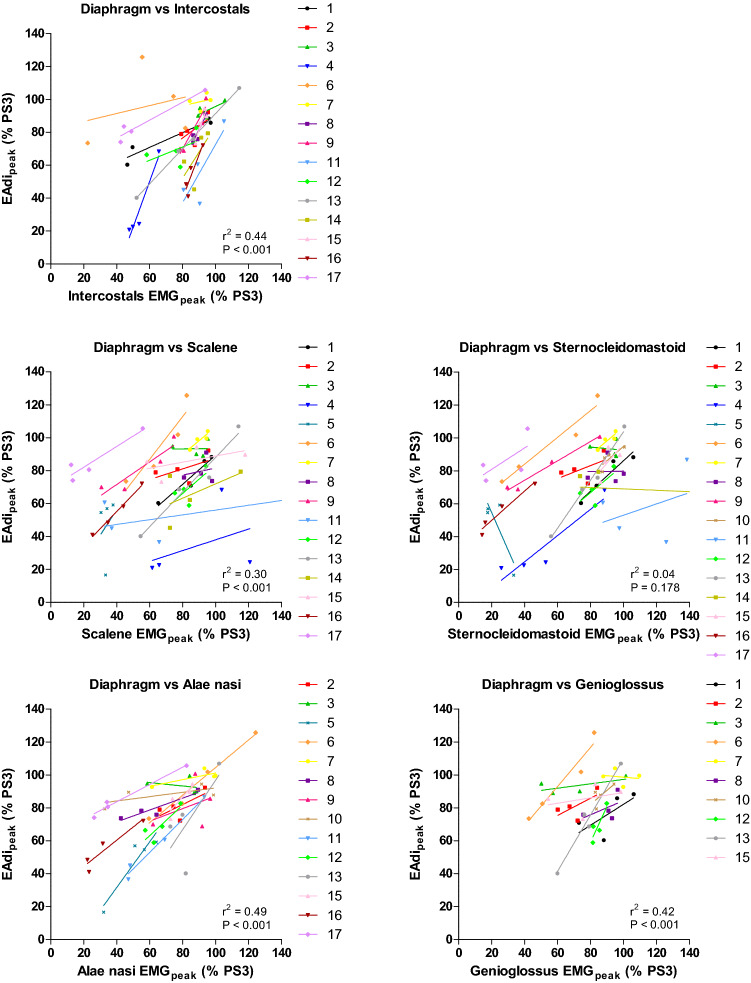
Relation between diaphragm and extradiaphragmatic inspiratory muscle activity for pressure support (PS) level 15, 12, 9 and 6 cmH_2_O, with peak electrical activity of the diaphragm (EAdi_peak_) and peak electromyography (EMG_peak_) normalized to muscle activity at PS level 3 cmH_2_O (% PS3). Each individual patient is depicted in a specific color and marker. The regression lines describing the relation on individual patient level are depicted, from which the high variability among patients is clear. 3 points are not shown (PS 6 patient 11 for scalene (scalene EMG_peak_ 298% PS 3 at EAdi_peak_ 87% PS 3) and PS 12 and 9 patient 14 for sternocleidomastoid (sternocleidomastoid EMG_peak_ 153 and 386% PS 3 at EAdi_peak_ 45 and 62% PS 3, respectively), as values were off scale. However, the regression lines shown include these data points

Calculation of correlation coefficients with repeated observations showed the highest positive correlation between EAdi_peak_ and alae nasi EMG_peak_ (*r*^2^ = 0.49; *P* < 0.001), with EMG_peak_ expressed as percentage relative to PS 3 cmH_2_O. This was followed by the parasternal intercostals EMG_peak_ (*r*^2^ = 0.44; *P* < 0.001) and genioglossus EMG_peak_ (*r*^2^ = 0.42; *P* < 0.001). A poor correlation was found between EAdi_peak_ and scalene EMG_peak_ (*r*^2^ = 0.30; *P* < 0.001), whereas no correlation was found between EAdi_peak_ and sternocleidomastoid EMG_peak_. On individual patient level, large differences were observed between the changes in diaphragm and extradiaphragmatic inspiratory muscle activity (Fig. 2).

Bland–Altman analyses (Additional file 1: Figure S4) showed that the bias between EAdi and surface EMG for each of the extradiaphragmatic inspiratory muscles is small; however, the 95% limits of agreement are large due to individual differences. Regardless of the level of support the limits of agreement remain large.

### Timing of extradiaphragmatic inspiratory muscle activity in relation to the diaphragm

**Fig. 3:**
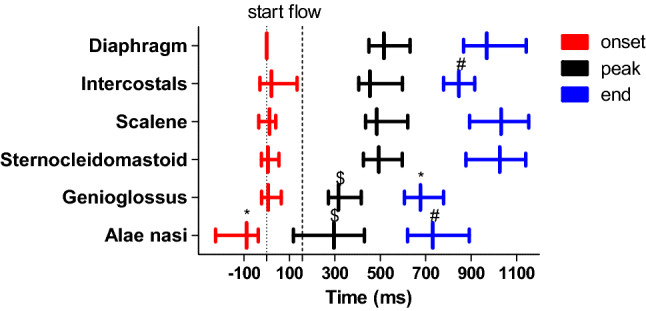
Relative onset, peak and termination times of extradiaphragmatic inspiratory muscle activity with respect to the onset time of diaphragm activity (time = 0 s). There are no differences in recruitment times between different PS levels; therefore, data are represented as median (interquartile range). For the alae nasi *N* = 61, genioglossus *N* = 43, sternocleidomastoid *N* = 69, scalene *N* = 70, parasternal intercostals *N* = 64 and diaphragm *N* = 72. *Significantly different with other muscles except alae nasi, ^$^significantly different with other muscles except alae nasi and genioglossus, ^#^significantly different with other muscles except intercostals and alae nasi (*P* < 0.05) . Inspiratory flow started significantly later than inspiratory muscle activity (*P* < 0.05)

Figure 3 shows the recruitment hierarchy of the diaphragm and extradiaphragmatic inspiratory muscles. Timing is relative to the onset of EAdi. Data were averaged per muscle for the different levels of support, because timing was not affected by the level of support.

Onset of alae nasi activity preceded the onset of all other muscles. Both peak and termination of electrical activity occurred earlier for both the alae nasi and genioglossus compared to the other muscles. In addition, parasternal intercostal activity terminated earlier than sternocleidomastoid, EAdi and scalene activity.

## Discussion

The main finding of the current study is that surface EMG of extradiaphragmatic inspiratory muscles does not reliably reflect activity of the diaphragm under different levels of inspiratory support. There is a moderate to low correlation and low agreement between changes in diaphragm and extradiaphragmatic inspiratory muscle activity in response to unloading of the respiratory muscles. Furthermore, there are notable differences in timing of activation between the diaphragm and extradiaphragmatic inspiratory muscles.

### Response of the extradiaphragmatic inspiratory muscles to different levels of inspiratory support

As expected, activity of the extradiaphragmatic inspiratory muscles increased in response to reducing level of assist on a group level. Our results are largely in accordance with previous studies in intubated patients. Schmidt et al. reported that parasternal intercostal, scalene and alae nasi activity increases when a low inspiratory PS level is applied as compared to a high PS level [26]. Cecchini et al. [28] showed that both NAVA and PS ventilation reduced alae nasi and scalene activity in proportion to the level of assistance. In addition, we found that this also holds for the genioglossus, despite that the endotracheal tube bypasses the upper airways. Remarkably, in most patients extradiaphragmatic inspiratory muscles remain active up to a PS level of 15 cmH_2_O. Brochard et al. [39] also demonstrated that the sternocleodomastoid muscle remains active at high inspiratory PS levels. These findings indicate high respiratory drive even at high levels of pressure support. High respiratory drive despite high levels of inspiratory assist may be explained by persistent abnormal arterial blood gas, feedback from afferents from the lung and chest wall or systemic inflammation (for review see [40]).

### Relationship between diaphragm and extradiaphragmatic inspiratory muscle activity

In the current study, we showed with repeated measures observation analysis that there are only moderate correlations between the changes in diaphragm and extradiaphragmatic inspiratory muscle activity (Fig. 2). Moreover, we demonstrate that there are large limits of agreement for all PS levels when comparing the changes in diaphragm and extradiaphragmatic inspiratory muscle activity (Additional file 1: Figure S4). For example, for the scalene and diaphragm, the 95% limits of agreement are between – 50 and + 50% PS 3 (normalized to muscle activity at PS level 3 cmH_2_O) for changes in surface EMG and EAdi_peak_ (Additional file 1: Figure S4). In clinical practice, such a measurement error is unacceptable, because this means, for example at an average EAdi_peak_ of 50% PS 3 (normalized to muscle activity at PS level 3 cmH_2_O) at PS 15, that there could be either no scalene activity or scalene EMG_peak_ could be doubled.

The relationship between diaphragm and extradiaphragmatic inspiratory muscle activity has been addressed previously. These studies reported that the recruitment pattern of extradiaphragmatic inspiratory muscles is comparable to the diaphragm in response to lower inspiratory support levels during noninvasive ventilation in healthy subjects [18, 37, 41], patients with chronic obstructive pulmonary disease (COPD) [18] and ventilated ICU patients [26, 28]. In contrast to our study, no correlation or agreement analysis were reported in most of these studies. In the study by Lin et al., there were also large limits of agreement between diaphragm and scalene muscle activity during noninvasive ventilation in COPD patients, whereas the parasternal intercostal muscles performed better [18]. COPD patients often have high levels of neural respiratory drive (for review see [42]) and thereby extradiaphragmatic inspiratory muscle activity is easier to detect with surface EMG.

Taken together, reducing inspiratory assist does not have a uniform effect on the diaphragm and extradiaphragmatic inspiratory muscles. Differences in responses among muscles may be partly explained by the fact that extradiaphragmatic inspiratory muscles are involved in other functions, such as patency of the upper airways, rotation of the head, flexion of the neck and stabilization of the trunk [20–25]. For the parasternal intercostal muscles the same motoneurons are depolarized during postural and inspiratory tasks; their output during inspiration is depending on the direction of the rotation of the trunk [23]. Furthermore, it has been shown that neural respiratory drive is not uniform in healthy subjects, and respiratory muscles recruit according to their mechanical advantage. In other words, respiratory muscles (or portions of muscles) with the greatest mechanical advantage for a specific task will be recruited earlier and to a larger extent [43, 44]. It seems plausible that the same is true in disease, which could result in differences in recruitment of extradiaphragmatic inspiratory muscles and the diaphragm with changes in ventilator support. Parthasarathy et al. [34] suggested such a hierarchy of respiratory muscle recruitment in patients failing a T-piece trial. In addition to the diaphragm and intercostal muscles, they demonstrated an immediate increase in sternocleidomastoid muscle activity with little change thereafter. The expiratory muscles are recruited relatively late during the T-piece trial: the largest increase in activity occurred only after 17–20 min. Finally, drive to the diaphragm may underestimate the true respiratory drive due to the contribution of the extradiaphragmatic inspiratory muscles, especially in critically ill patients there may be a discrepancy. Respiratory drive can be higher in critically ill patients not only due to the load on the respiratory muscles, but also due to metabolic acidosis and hypoxemia, brain, lung or chest wall pathologies (for review see [40]).

### Timing of extradiaphragmatic inspiratory muscle activity

In healthy subjects there is a clear hierarchy with respect to respiratory muscle recruitment [45, 46]. For example, upper airway muscles recruit ± 100 ms before the diaphragm recruits in healthy subjects [21]. We studied more different muscles and applied different PS levels as compared to previous studies [26, 27]. The alae nasi recruited earlier as compared to other extradiaphragmatic inspiratory muscles (132–172 ms) and the diaphragm (122 ms). We found no differences in timing of the extradiaphragmatic inspiratory muscles between ventilator settings. These results were expected based on previous studies [26, 27]. Schmidt et al. [27] observed that recruitment onset times were similar among the scalene, sternocleidomastoid and genioglossus in mechanically ventilated patients.

### Practical limitations of surface EMG

In addition to the poor correlations and low agreement between changes in activity between the diaphragm and extradiaphragmatic inspiratory muscles in response to unloading, there are practical issues that limit the applicability of surface EMG to monitor drive to the diaphragm. First, we found that in several patients no muscle activity could be detected from the genioglossus, alae nasi, parasternal intercostals and scalene during the whole study protocol. This could be the result from real inactivity of the muscles or low signal-to-noise ratio. Second, surface EMG is vulnerable to noise (e.g. electromagnetic noise) and artifacts (e.g. due to movement), these cannot be avoided, but the effects can be minimized in the preprocessing and analyzing process [47]. Third, the technique is technically challenging in obese patients, restless patients, or patients with diaphoresis. Note that data used in the current study were highly selected. Large periods of data were not useful to study breathing activity because patients were moving their head or body resulting in non-breathing-related muscle activity.

### Study limitations

The current study has some limitations. First, we did not measure force, only EMG as a measure for respiratory drive. Respiratory drive can be evaluated at the bedside by several methods (for recent review see [40]). The only method to measure the contribution of extradiaphragmatic inspiratory muscles to respiration is by surface EMG. Therefore, we wanted to evaluate the recruitment pattern of extradiaphragmatic inspiratory muscles with respect to the diaphragm. Second, we did not measure surface EAdi. Bellani et al. [17] demonstrated that surface EAdi correlated well with EAdi, although there was a high variability in the slopes between patients. They showed that respiratory effort could be calculated from surface EAdi, but when comparing surface EAdi with esophageal pressure to compute muscular pressure, this resulted in low bias but large limits of agreement. Calculation of effort from both the diaphragm and extradiaphragmatic inspiratory muscles did not result in an improved estimation of respiratory effort as compared to EAdi or surface EAdi. Third, the study was not blinded. The signals were analyzed offline, only periods to be analyzed were selected manually, while the rest of the analysis was performed automatically using a custom-written script. Therefore, the unblinded nature is unlikely to affect the results. Fourth, accuracy of calculating recruitment times depends on the manner in which the threshold for muscle activity is determined, and also on the noise level. Therefore, not only relative onset times were computed, but also peak and termination times. For all three parameters the same trends were found and recruitment times were in the same range.

## Conclusions

In the current study, we investigated potential surrogate markers of diaphragm activity. We demonstrate that extradiaphragmatic inspiratory muscle activity increases in response to lower inspiratory support levels. However, we found moderate correlations and low agreement between changes in diaphragm activity and extradiaphragmatic inspiratory muscle activity. Therefore, it is concluded that monitoring of respiratory drive is not feasible using extradiaphragmatic inspiratory muscle activity. We demonstrate that the magnitude and timing of muscle activity differ among inspiratory muscles, making it very cumbersome to monitor patient–ventilator interactions.

## Supplementary information

**Additional file 1.** Additional figures.

## Data Availability

The datasets used and analyzed during the current study are available from the corresponding author on reasonable request.

## Abbreviations

AAA: Abdominal aortic aneurysm
ARDS: Acute respiratory distress syndrome
COPD: Chronic obstructive pulmonary disease
EAdi: Electrical activity of the diaphragm
EMG: Electromyography
MV: Mechanical ventilation
NAVA: Neurally adjusted ventilatory assist
PEEP: Positive end-expiratory pressure
PJP: Pneumocystic jirovecii pneumonia
PS: Pressure support
RMS: Root mean square

## References

[1] Dres M, Goligher EC, Heunks LMA, Brochard LJ (2017). Critical illness-associated diaphragm weakness. Intensive Care Med.

[2] Hooijman PE, Beishuizen A, Witt CC, de Waard MC, Girbes AR, Spoelstra-de Man AM (2015). Diaphragm muscle fiber weakness and ubiquitin-proteasome activation in critically ill patients. Am J Respir Crit Care Med.

[3] Jaber S, Petrof BJ, Jung B, Chanques G, Berthet JP, Rabuel C (2011). Rapidly progressive diaphragmatic weakness and injury during mechanical ventilation in humans. Am J Respir Crit Care Med.

[4] Hudson MBSAJ, Nelson WB, Bruells CS, Levine S, Powers SK (2012). Both high level pressure support ventilation and controlled mechanical ventilation induce diaphragm dysfunction and atrophy. Crit Care Med.

[5] Ebihara SHSNA, Danialou G, Cho W, Gottfried SB, Petrof BJ (2002). Mechanical ventilation protects against diaphragm injury in sepsis. Am J Respir Crit Care Med.

[6] Goligher ECFE, Herridge MS, Murray A, Vorona S, Brace D, Rittayamai N, Lanys A, Tomlinson G, Singh JM, Bolz S, Rubenfeld GD, Kavanagh BP, Brochard LJ, Ferguson ND (2015). Evolution of diaphragm thickness during mechanical ventilation: impact of inspiratory effort. Am J Respir Crit Care Med.

[7] Levine S, Nguyen T, Taylor N, Friscia ME, Budak MT, Rothenberg P, Zhu BAJ, Sachdeva R, Sonnad S, Kaiser LR, Rubinstein NA, Powers SK, Shrager JB (2008). Rapid disuse atrophy of diaphragm fibers in mechanically ventilated humans. N Engl J Med.

[8] Reid WDHJ, Bryson S, Walker DC, Belcastro AN (1994). Diaphragm injury and myofibrillar structure induced by resistive loading. J Appl Physiol.

[9] Jiang TRWD, Road JD (1998). Delayed diaphragm injury and diaphragm force production. Am J Respir Crit Care Med.

[10] Bellani G, Pesenti A (2014). Assessing effort and work of breathing. Curr Opin Crit Care.

[11] Doorduin J, van Hees HW, van der Hoeven JG, Heunks LM (2013). Monitoring of the respiratory muscles in the critically ill. Am J Respir Crit Care Med.

[12] Heunks LM, Doorduin J, van der Hoeven JG (2015). Monitoring and preventing diaphragm injury. Curr Opin Crit Care.

[13] Goligher EC, Brochard LJ, Reid WD, Fan E, Saarela O, Slutsky AS (2019). Diaphragmatic myotrauma: a mediator of prolonged ventilation and poor patient outcomes in acute respiratory failure. Lancet Respir Med..

[14] Akoumianaki E, Maggiore SM, Valenza F, Bellani G, Jubran A, Loring SH (2014). The application of esophageal pressure measurement in patients with respiratory failure. Am J Respir Crit Care Med.

[15] Mauri T, Yoshida T, Bellani G, Goligher EC, Carteaux G, Rittayamai N (2016). Esophageal and transpulmonary pressure in the clinical setting: meaning, usefulness and perspectives. Intensive Care Med.

[16] Bellani G, Mauri T, Coppadoro A, Grasselli G, Patroniti N, Spadaro S (2013). Estimation of patient’s inspiratory effort from the electrical activity of the diaphragm. Crit Care Med.

[17] Bellani G, Bronco A, Arrigoni Marocco S, Pozzi M, Sala V, Eronia N (2018). Measurement of diaphragmatic electrical activity by surface electromyography in intubated subjects and its relationship with inspiratory effort. Respir Care..

[18] Lin L, Guan L, Wu W, Chen R (2019). Correlation of surface respiratory electromyography with esophageal diaphragm electromyography. Respir Physiol Neurobiol.

[19] Dres M, Dube BP, Goligher EC, Vorona S, Demiri S, Brochard LJ, Similowsky T, Demoule A (2018). Intercostal muscle ultrasound: a feasibility and physiological study in mechanically ventilated patients [abstract]. Am J Respir Crit Care Med.

[20] Oppersma E, Doorduin J, van der Heijden EHRM, van der Hoeven JG, Heunks LMA (2013). Noninvasive ventilation and the upper airway: should we pay more attention?. Crit Care..

[21] Strohl KPHMJ, Hallett M, Saunders NA, Ingram RH (1980). Activation of upper airway muscles before onset of inspiration in normal humans. J Appl Physiol.

[22] Saboisky JP, Jordan AS, Eckert DJ, White DP, Trinder JA, Nicholas CL (2010). Recruitment and rate-coding strategies of the human genioglossus muscle. J Appl Physiol.

[23] Hudson AL, Butler JE, Gandevia SC, De Troyer A (2010). Interplay between the inspiratory and postural functions of the human parasternal intercostal muscles. J Neurophysiol.

[24] Hudson AL, Butler JE, Gandevia SC, De Troyer A (2011). Role of the diaphragm in trunk rotation in humans. J Neurophysiol.

[25] Moore KL, Dalley AF, Agur AM. Chapter 8 Neck. Clinically oriented anatomy. 5th ed. 2006. p. 1052–78.

[26] Schmidt M, Kindler F, Gottfried SB, Raux M, Hug F, Similowski T (2013). Dyspnea and surface inspiratory electromyograms in mechanically ventilated patients. Intensive Care Med.

[27] Schmidt MCL, Hug F, Demoule A, Similowski T (2011). Surface electromyogram of inspiratory muscles: a possible routine monitoring tool in the intensive care unit. Br J Anaesth.

[28] Cecchini JSM, Demoule A, Similowski T (2014). Increased diaphragmatic contribution to inspiratory effort during neurally adjusted ventilatory assistance versus pressure support. Anesthesiology.

[29] Doorduin J, Nollet JL, Roesthuis LH, van Hees HW, Brochard LJ, Sinderby CA (2017). Partial neuromuscular blockade during partial ventilatory support in sedated patients with high tidal volumes. Am J Respir Crit Care Med.

[30] Doorduin J, Roesthuis LH, Jansen D, van der Hoeven JG, van Hees HWH, Heunks LMA (2018). Respiratory muscle effort during expiration in successful and failed weaning from mechanical ventilation. Anesthesiology.

[31] Doorduin J, Sinderby CA, Beck J, van der Hoeven JG, Heunks LM (2015). Assisted ventilation in patients with acute respiratory distress syndrome: lung-distending pressure and patient–ventilator interaction. Anesthesiology.

[32] Doorduin J, Sinderby CA, Beck J, van der Hoeven JG, Heunks LMA (2014). Automated patient–ventilator interaction analysis during neurally adjusted non-invasive ventilation and pressure support ventilation in chronic obstructive pulmonary disease. Crit Care..

[33] Beck J, Gottfried SB, Navalesi P, Skrobik Y, Comtois N, Rossini M (2001). Electrical activity of the diaphragm during pressure support ventilation in acute respiratory failure. Am J Respir Crit Care Med.

[34] Parthasarathy S, Jubran A, Laghi F, Tobin MJ (2007). Sternomastoid, rib cage, and expiratory muscle activity during weaning failure. J Appl Physiol.

[35] Falla DDAP, Rainoldi A, Merletti R, Jull G (2002). Location of innervation zones of sternocleidomastoid and scalene muscles—a basis for clinical and research electromyography applications. Clin Neurophysiol.

[36] Zhan C, Yeung LF, Yang Z (2010). A wavelet-based adaptive filter for removing ECG interference in EMGdi signals. J Electromyogr Kinesiol.

[37] Chiti L, Biondi G, Morelot-Panzini C, Raux M, Similowski T, Hug F (2008). Scalene muscle activity during progressive inspiratory loading under pressure support ventilation in normal humans. Respir Physiol Neurobiol.

[38] Bland J M., Altman D. G (1995). Statistics notes: Calculating correlation coefficients with repeated observations: Part 1--correlation within subjects. BMJ.

[39] Brochard LHA, Lorino H, Lemaire F (1989). Inspiratory pressure support prevents diaphragmatic fatigue during weaning from mechanical ventilation. Am Rev Respir Dis.

[40] Vaporidi K, Akoumianaki E, Telias I, Goligher EC, Brochard L, Georgopoulos D. Respiratory drive in critically ill patients: pathophysiology and clinical implications. Am J Respir Crit Care Med. 2019. 10.1164/rccm.201903-0596SO 31437406

[41] Hug F, Raux M, Morelot-Panzini C, Similowski T (2011). Surface EMG to assess and quantify upper airway dilators activity during non-invasive ventilation. Respir Physiol Neurobiol.

[42] McKenzie DK, Butler JE, Gandevia SC (2009). Respiratory muscle function and activation in chronic obstructive pulmonary disease. J Appl Physiol.

[43] Butler JE (2007). Drive to the human respiratory muscles. Respir Physiol Neurobiol.

[44] Hudson AL, Gandevia SC, Butler JE (2007). The effect of lung volume on the co-ordinated recruitment of scalene and sternomastoid muscles in humans. J Physiol.

[45] De Troyer AKPA, Wilson TA (2005). Respiratory action of the intercostal muscles. Physiol Rev.

[46] Saboisky JP, Gorman RB, De Troyer A, Gandevia SC, Butler JE (2007). Differential activation among five human inspiratory motoneuron pools during tidal breathing. J Appl Physiol.

[47] Hug F (2011). Can muscle coordination be precisely studied by surface electromyography?. J Electromyogr Kinesiol.

