# Supplementary material for: Recruitment pattern of the diaphragm and extradiaphragmatic inspiratory muscles in response to different levels of pressure support
Source: Ann Intensive Care. 2020 May 29;10:67. doi: 10.1186/s13613-020-00684-6 (PMC7256918; doi:10.1186/s13613-020-00684-6)
Supplement: Supplementary file 1 — Additional file 1. Additional figures. [file 13613_2020_684_MOESM1_ESM.docx]

**Recruitment pattern of the diaphragm and extradiaphragmatic inspiratory muscles in response to different levels of pressure support**

**Additional file 1**

L.H. Roesthuis M.Sc.^1^; J.G. van der Hoeven M.D., Ph.D.^1^; H.W.H. van Hees Ph.D.^2^;
W-J. M. Schellekens M.D., Ph.D.^3^; J. Doorduin Ph.D.^4^; L.M.A. Heunks M.D., Ph.D.^5^

^1^ Department of Intensive Care Medicine, Radboud University Medical Center, Nijmegen, The Netherlands; ^2^ Department of Pulmonary Diseases, Radboud University Medical Center, Nijmegen, The Netherlands; ^3^ Department of Anesthesiology, UMC Utrecht, Utrecht, The Netherlands; ^4^ Donders Institute for Brain, Cognition and Behaviour, Department of Neurology, Radboud University Medical Center, Nijmegen, The Netherlands; ^5^ Department of Intensive Care Medicine, Amsterdam UMC, location VUmc, The Netherlands

**FIGURE LEGENDS**

**Figure S1** Processing of the electromyographic (EMG) signal. **A.** Raw EMG signal with cardial artefacts. **B.** EMG signal after removing cardial artefacts. **C.** The root mean square (RMS) was determined and an envelope could be constructed of the signal. A threshold was visually determined to calculate the onset of muscle activity. Parameters to quantify magnitude of muscle activity (EMG_peak_) and recruitment times (onset, peak and end) could be determined from the envelope of each inspiratory effort.

**Figure S2** Individual responses of the diaphragm and extradiaphragmatic inspriatory muscles on reducing pressure support (PS) level. There is a high variability among patients and inspiratory muscles. Note the outliers for the scalene and sternocleidomastoid, the five minute recording from this study step in these patients was highly variable (from almost no activity to high magnitude of activity), making it difficult to select a representative period suitable for data analysis. Peak electromyography (EMG_peak_) is normalized to muscle activity at PS level 3 cmH_2_O (% PS3).

**Figure S3** Extradiaphragmatic inspiratory muscle activity increases when lower inspiratory support levels are applied (*P* < 0.05). The area under the EMG signal (EMG_AUC/min_) values are normalized to activity at 3 cmH_2_O pressure support (PS). Values are represented as median (interquartile range). The number of subjects in which EMG parameters could be determined differed per muscle and PS level. Globally, for the alae nasi N = 13, genioglossus N = 9, scalene N = 15, sternocleidomastoid 15 and diaphragm N = 16 and parasternal intercostals N = 15.

* = significant difference with PS 3, $ = significant difference with PS 6 (*P* < 0.05)

**Figure S4** Bland-Altman analysis in which electrical activity of the diaphragm is compared to extradiaphragmatic inspiratory muscle activity (EMG_peak_) for pressure support (PS) levels 15 (red square), 12 (black closed circle), 9 (green triangle) and 6 cmH_2_O (blue triangle upside-down). There are large differences between both measuring methods for each of the extradiaphragmatic inspiratory muscles, also there is a high variability among individual patients. The limits of agreement are large regardless of the inspiratory support level. EMG_peak_ is normalized to muscle activity at PS level 3 cmH_2_O (% PS3).

**Figure S1**

**
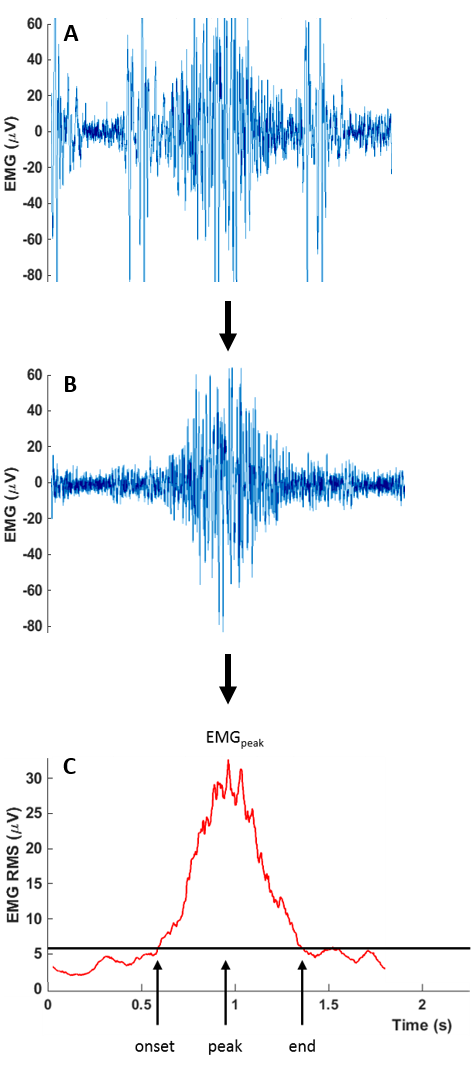
**

**Figure S2**

**Figure S3**

**Figure S4**
